# Cerebrospinal fluid lipid profiles as exploratory biomarkers for pediatric meningitis: a proof-of-concept case series

**DOI:** 10.3389/fncel.2026.1816621

**Published:** 2026-05-29

**Authors:** Feng Tang, Changzhen Li, Ye Zeng, Wanjun Luo, Lei Xi, Xiaomei Wang

**Affiliations:** 1 Department of Laboratory Medicine, Wuhan Children’s Hospital (Wuhan Maternal and Child Healthcare Hospital), Tongji Medical College, Huazhong University of Science & Technology, Wuhan, China; 2 Hospital-Acquired Infection Control Department, Wuhan Children’s Hospital (Wuhan Maternal and Child Healthcare Hospital), Tongji Medical College, Huazhong University of Science & Technology, Wuhan, China

**Keywords:** cerebrospinal fluid, differential diagnosis, lipid metabolomics, mass spectrometry, pediatric meningitis

## Abstract

**Objectives:**

To investigate the cerebrospinal fluid (CSF) lipid metabolite profiles of pediatric patients with purulent meningitis (PM) or viral meningitis (VM) and to explore their differential diagnostic potential.

**Methods:**

In this proof-of-concept case series, 13 CSF samples from 10 pediatric patients were analyzed by lipidomics, distributed across four sample-level analytical groups: acute-phase PM (PM_A, *n* = 3) and recovery-phase PM (PM_R, *n* = 3) from 3 PM patients with paired sampling, acute-phase VM (VM_A, *n* = 3) from 3 VM patients, and non-meningitic controls (N, *n* = 4). Lipid separation and detection were performed by UPLC-MS/MS on a Q Exactive high-resolution mass spectrometer, and data were processed using LipidSearch v.4.1.

**Results:**

Compared with the N group, the PM_A group displayed exploratory lipid alterations with 30 increased and 45 decreased metabolites, predominantly involving sphingolipid (SPH), dihexosylceramide (Hex2Cer), monohexosylceramide (Hex1Cer), lysophosphatidylcholine (LPC), and acylcarnitine (AcCa) species. Elevated Hex2Cer combined with reduced SPH represented a distinctive exploratory PM_A pattern. Receiver operating characteristic (ROC) analysis identified LPC(20:3) and Hex2Cer(d42:3) as exploratory candidate features for distinguishing PM_A from N (apparent AUC = 0.833 for each marker; leave-one-out jackknife AUC range 0.750–1.000, mean 0.833). Multi-feature composite AUCs were not reported because, at *n* = 7, any composite would be highly susceptible to overfitting. The VM_A group showed limited deviation from N (4 increased, 6 decreased metabolites) but differed from PM_A in 32 increased and 15 decreased metabolites, primarily within SPH, phosphatidylserine (PS), MePC, LPC, and AcCa subclasses. SM(d35:2) emerged as an exploratory candidate distinguishing VM_A from both N and PM_A, but at *n* = 3 vs. 4 the apparent AUC = 1.000 is statistically expected for some of the 344 screened metabolites by chance alone, and the candidate is therefore hypothesis-generating only.

**Conclusion:**

Cerebrospinal fluid lipidomics revealed disease-stage-specific alterations in pediatric PM and VM. These findings should be regarded as hypothesis-generating in this small proof-of-concept cohort and require validation in larger pediatric studies.

## Introduction

Meningitis, an inflammation of the meninges surrounding the brain and spinal cord, presents a significant global health burden, particularly affecting children (GBD 2019 Diseases and Injuries Collaborators, 2020; Basatemur, 2023). Meningitis can be bacterial or aseptic, with viral, tuberculous, and other pathogens accounting for the latter (Wilson et al., 2019). The current diagnostic paradigm relies on patient history, clinical presentation, imaging, and sequential laboratory examinations. However, in pediatric practice, overlapping features of infectious and non-infectious origins and frequently non-specific symptoms complicate differentiation (Wilson et al., 2019). In aseptic meningitis in particular, conventional cerebrospinal fluid (CSF) culture and routine laboratory tests often fail to confirm the etiology. Novel CSF biomarkers for different meningitis types are therefore needed to support prompt diagnosis and management.

Cerebrospinal fluid is largely water but also contains enzymes, metal ions, micronutrients, neurotransmitters, amino acids, glucose, carbohydrates, short-chain fatty acids, alcohols, peptides, and proteins. Metabolite profiles in CSF change with neuroinflammation, regardless of bacterial or aseptic origin (Yan et al., 2021). Different meningitis types display varied clinical manifestations and CSF compositional changes (Shukla et al., 2017; Plaatjie et al., 2024). Analyzing CSF metabolites, interpreting the resulting data, and understanding the underlying biochemical changes are therefore important for biomarker discovery and targeted treatment (Yan et al., 2021). While conventional CSF culture and microscopy remain essential, recent advances in metabolomics–particularly UPLC-MS/MS-based approaches–offer promising tools to characterize the pathophysiology of meningitis. Recent CSF lipidomics and metabolomics studies have started to identify lipid and metabolite signatures that distinguish bacterial from viral CNS infections and from non-inflammatory controls (de Araujo et al., 2020; Al-Mekhlafi et al., 2021, 2023; Ratuszny et al., 2019; Neu et al., 2024). Comprehensive reviews summarizing CSF lipidomics and metabolomics across viral and bacterial CNS infections (Plaatjie et al., 2024) provide a useful contextual framework, and CSF lipid profiling has likewise been explored in non-infectious neurological diseases such as multiple sclerosis (Nogueras et al., 2019), Rett syndrome (Zandl-Lang et al., 2022), and neurosyphilis (Liu et al., 2023), as well as in postoperative neuroinflammation (Terrando et al., 2021). In a pediatric cohort, gas-chromatography-based CSF fatty-acid profiling has previously distinguished purulent from non-purulent meningitis on the basis of oleic-acid and ω-3 PUFA content (Ekhtiyari et al., 2017), and an LC-MS metabolomics pilot study in pediatric intracranial bacterial infection further confirmed broad CSF metabolic remodeling in this population (Wang et al., 2021). Most of this evidence, however, is based on adult cohorts, leaving a knowledge gap regarding pediatric CSF lipidomics.

Intriguingly, in a recent pediatric case of purulent meningitis (PM, also referred to as bacterial meningitis), we observed a distinctive CSF appearance with a strawberry-milk-like yellow hue and turbidity, suggesting a possible link between CSF lipid components and meningitis. In the present proof-of-concept case series, we performed a comprehensive lipidomic analysis of CSF in pediatric patients with PM and viral meningitis (VM). Our objective was to explore disease- and disease-stage-specific lipid features in pediatric meningitis and to generate hypotheses for future biomarker validation. Given the small sample size, all candidate features should be interpreted as exploratory.

## Materials and methods

### Study design and sample collection

This proof-of-concept case series was conducted at Wuhan Children’s Hospital and included 10 pediatric patients (Table 1) classified into three diagnostic categories based on clinical evaluation, imaging, microbiological tests, and routine CSF laboratory results: purulent meningitis (PM), viral meningitis (VM), and non-meningitic controls (N). N samples were obtained from pediatric patients in whom meningitis was clinically suspected but not subsequently confirmed by laboratory and imaging evidence. The 10 enrolled patients comprised 3 PM patients (including the index case described in detail in the section “Results”), 3 VM patients, and 4 N patients. A total of 13 CSF samples were analyzed by lipidomics across four sample-level analytical groups: PM_A (acute phase, *n* = 3), PM_R (recovery phase, *n* = 3), VM_A (acute phase, *n* = 3), and N (*n* = 4). Each PM patient contributed both an acute-phase and a recovery-phase CSF sample (paired sampling, 2 samples per patient), each VM patient contributed one acute-phase CSF sample, and each N patient contributed one CSF sample. Paired acute- and recovery-phase CSF samples were available only for PM patients; VM patients contributed only acute-phase CSF samples, and therefore a separate VM_R analytical group was not generated. The collected samples were stored at −80 °C until further analysis.

**TABLE 1:** Acute-phase or diagnostic clinical manifestations and routine CSF laboratory findings of the 10 pediatric patients enrolled in the case series (3 PM, 3 VM, 4 N).

		PM			VM			N			
Category	Case	1	2	3	4	5	6	7	8	9	10
Clinical symptoms and signs	Gender	F	M	M	F	M	F	F	F	F	F
	Age	1y1m	1d	7d	2y9m	3y2m	5y11m	1y10m	2y3m	2y6m	2y
	Hospital stay	44d	21d	41d	4d	12d	12d	3d	5d	1d	18d
	Fever	√		√	√	√	√		√		
	Hyperspasmia	√			√						
	Headache					√					
	Drowsiness	√		√		√			√		
	Vomit					√	√				
	Consciousness						√				
	AF tension↑		√								
	Limb muscle tone↑		√								
	Neck rigidity	√		√		√	√				
	Kerning sign										
	Brudzinski sign	√									
Laboratory parameters of CSF	Color	CY	Y	Y	W	CL	CL	CL	CL	CL	CL
	Turbidity	Turbid	Turbid	Turbid	Turbid	s-turbid	s-turbid	Clear	Clear	Clear	Clear
	WBC	8323	10267	23959	43629	28	136	0	28	0	0
	NEU	6742	9548	20365	31849	1	24	/	0	/	/
	LYM	8.32	103	958	436	23	102	/	22	/	/
	MON	1498	616	2635	11343	4	10	/	6	/	/
	CL	111.9	106.3	106.5	118.3	126.3	118.7	125.4	129.2	124.7	122.9
	GLU	1.63	0.02	2.51	0.04	2.75	3.41	3.18	3.54	3.25	3.04
	TP	4.27	13.27	2.18	1.6	0.14	0.52	0.16	0.33	0.14	0.2
	CSF culture	Pae			Spn						

Cell counts: WBC, white blood cells; NEU, neutrophils; LYM, lymphocytes; MON, monocytes (×106/L). RBC, red blood cells (×10^9^/L). CL, chloride; GLU, glucose; TC, total cholesterol; TG, triglyceride; HDL, high-density lipoprotein; LDL, low-density lipoprotein (mmol/L); TP, total protein (g/L). AF, anterior fontanel; CY, creamy yellow; Y, yellow; W, white; CL (color row), colorless; s-turbid, slightly turbid; Pae, *Pseudomonas aeruginosa*; Spn, *Streptococcus pneumoniae*; √, presence of corresponding symptom or sign. Table 1 summarizes acute-phase or diagnostic clinical data for the 10 enrolled pediatric patients comprising 3 PM patients (including the index case described in detail in the section “Results”), 3 VM patients, and 4 non-meningitic control (N) patients. The CSF lipidomic analysis included 13 CSF samples distributed across four sample-level analytical groups: PM_A (*n* = 3) and PM_R (*n* = 3), with each of the 3 PM patients contributing one acute-phase and one recovery-phase CSF sample; VM_A (*n* = 3), with each of the 3 VM patients contributing one acute-phase CSF sample; and N (*n* = 4), with each of the 4 N patients contributing one CSF sample.

### Laboratory examination of CSF

After concentrated centrifugation (CYTOSPIN4, Thermo Fisher Scientific, USA), CSF samples were fixed and immersed in Harris hematoxylin for 1–2 min for light staining, then rinsed with tap water, transferred to 70% alcohol for 5 s, immersed in Sudan III stain for approximately 30 min at 56 °C, washed in 70% alcohol for 5–10 s, and rinsed with distilled water before microscopic observation. Cell counts and biochemical analyses were performed using an XN-3000 automatic blood cell analyzer (Sysmex, Japan) and a COBAS C702 automatic biochemical analyzer (Roche, Switzerland) in routine fluid mode. Test parameters included red blood cell counts, white blood cell counts, white blood cell classifications, chloride, glucose, protein, total cholesterol (TC), triglyceride (TG), high-density lipoprotein (HDL), and low-density lipoprotein (LDL). For bacterial culture, CSF samples were inoculated onto blood, MacConkey, or chocolate agar plates and incubated at 37 °C for 20 h; suspected colonies were identified using matrix-assisted laser desorption/ionization time-of-flight mass spectrometry.

### Lipid metabolite analysis with UPLC-MS

A Waters UPLC I-Class Plus system coupled with a Q Exactive high-resolution mass spectrometer (Thermo Fisher Scientific, USA) was used for metabolite separation and detection. Chromatographic separation employed a CSH C18 column (1.7 μm, 2.1 mm × 100 mm; Waters). Mobile phase A consisted of 60% acetonitrile in water with 10 mM ammonium formate and 0.1% formic acid, and mobile phase B consisted of 90% isopropanol with 10% acetonitrile, 10 mM ammonium formate, and 0.1% formic acid. Column temperature was 55 °C. The gradient elution program was: 40% B at 0 min, 40%–43% B over 0–2 min, 43%–50% B over 2–2.1 min, 50%–54% B over 2.1–7 min, 54%–70% B over 7–7.1 min, 70%–99% B over 7.1–13 min, 99% B over 13–13.1 min, and 99%–40% B over 13.1–15 min. Flow rate was 0.4 mL/min and injection volume was 5 μL. For mass spectrometry, full-scan spectra were acquired in the range 70–1050 m/z at a resolution of 70,000 with an automatic gain control (AGC) target of 3e6 and a maximum ion injection time of 100 ms. The top three precursor ions were selected for MS/MS fragmentation at a resolution of 17,500, AGC target 1e5, maximum injection time 50 ms, and stepped normalized collision energy of 15, 30, and 45 eV. Electrospray ionization parameters were: sheath gas flow rate 40, auxiliary gas flow rate 10, spray voltage 3.80 kV (positive) and 3.20 kV (negative), capillary temperature 320 °C, and auxiliary gas heater temperature 350 °C.

### Metabolite identification

Mass-spectrometry data were imported into LipidSearch v.4.1 (Thermo Fisher Scientific, USA) for lipid identification and quantitative matrix generation, followed by additional data processing. The CSF lipidome workflow framework is consistent with previously established LC-HRMS approaches for human CSF lipid annotation (Seyer et al., 2016).

### Statistical analysis

Because the present study is an exploratory proof-of-concept case series with a very limited sample size, all comparative analyses were considered descriptive and hypothesis-generating rather than confirmatory. Per-metabolite analysis was performed for each of the 344 identified lipid metabolites and for the 21 lipid sub-class summaries. For each comparison (PM_A vs. N, PM_R vs. N, VM_A vs. N, PM_R vs. PM_A, and VM_A vs. PM_A), the fold change (FC) was calculated as the ratio of the mean peak intensity of the first group to that of the second group, and a nominal exploratory *P*-value was calculated by Welch’s *t*-test on log10-transformed sample-level peak intensities (which handles unequal variances and is robust to the small group sizes of *n* = 3 to *n* = 4). An exploratory screening criterion of FC ≥ 1.2 (or ≤1/1.2) and nominal *P* < 0.05 was used to identify candidate lipid features for descriptive reporting and is the criterion underlying the counts of differentially regulated metabolites reported in the Results.

To address the multiplicity inherent in screening 344 metabolites, Benjamini–Hochberg false discovery rate (BH-FDR) *Q*-values were calculated within each comparison and are reported in the supplementary per-metabolite statistics table. Because of the very small sample size, the present study uses nominal exploratory *P*-values as the primary reporting metric; BH-FDR *Q*-values are reported in parallel for transparency. Candidate features that survive Q < 0.10 within the PM_A vs. N comparison are highlighted in the Results. For the lipid-class summaries, the mean peak intensity of each lipid sub-class was computed as the sum of all metabolite peak intensities within that sub-class for each sample, followed by averaging across samples within each analytical group. Lipid-class FC and Welch’s nominal *P*-values relative to N are reported in Supplementary Table 1. The diagnostic potential of candidate features was characterized by the apparent within-cohort area under the receiver operating characteristic curve (AUC). Internal stability of the candidate features was explored using a leave-one-out (LOO) jackknife procedure, in which the AUC was recalculated after each sample was removed in turn, and the mean and range of jackknife AUCs are reported. Multi-feature composite scores were not computed for ROC analysis because, with the very small sample size (7 samples in the PM_A vs. N comparison and 7 in the VM_A vs. N comparison), any data-driven combination would be highly susceptible to overfitting; we therefore restrict ROC reporting to single-feature analyses with jackknife uncertainty. No independent validation cohort was available; thus, the AUC values reported here represent within-cohort performance only and require external validation in larger pediatric cohorts.

## Results

### Brief case presentation of PM with fatty cerebrospinal fluid

On February 2nd, 2022, a child aged 1 year and 1 month was transferred to the intensive care unit of Wuhan Children’s Hospital after prior treatment at an outside hospital. Her guardian reported intermittent fever for over half a month and intermittent seizures for 11 days before transfer. CSF examination performed at the outside hospital on January 23rd showed marked pleocytosis and elevated protein levels, and CSF culture and next-generation sequencing on January 28th identified *Streptococcus pneumoniae*. Physical examinations on admission to our hospital revealed an open anterior fontanel (approximately 0.3 cm × 0.3 cm) with slightly increased tension, and pupils were round and equally sized at 2.5 mm with sluggish pupillary light reflexes. The patient exhibited somnolence, a positive Babinski sign, and neck stiffness, with neck flexion less than one fingerbreadth below the chin (Table 1). Cerebral computed tomography indicated subarachnoid hemorrhage, brain edema, and hydrocephalus involving the basal ganglia. The child was diagnosed with purulent meningitis, cerebral abscess, and hydrocephalus. Emergency bilateral ventricular puncture and drainage were performed, followed by treatment with ceftriaxone, linezolid, dopamine, midazolam (maximum infusion rate 5 μg/kg/min), and methylprednisolone, achieving effective control of the condition.

**FIGURE 1:**
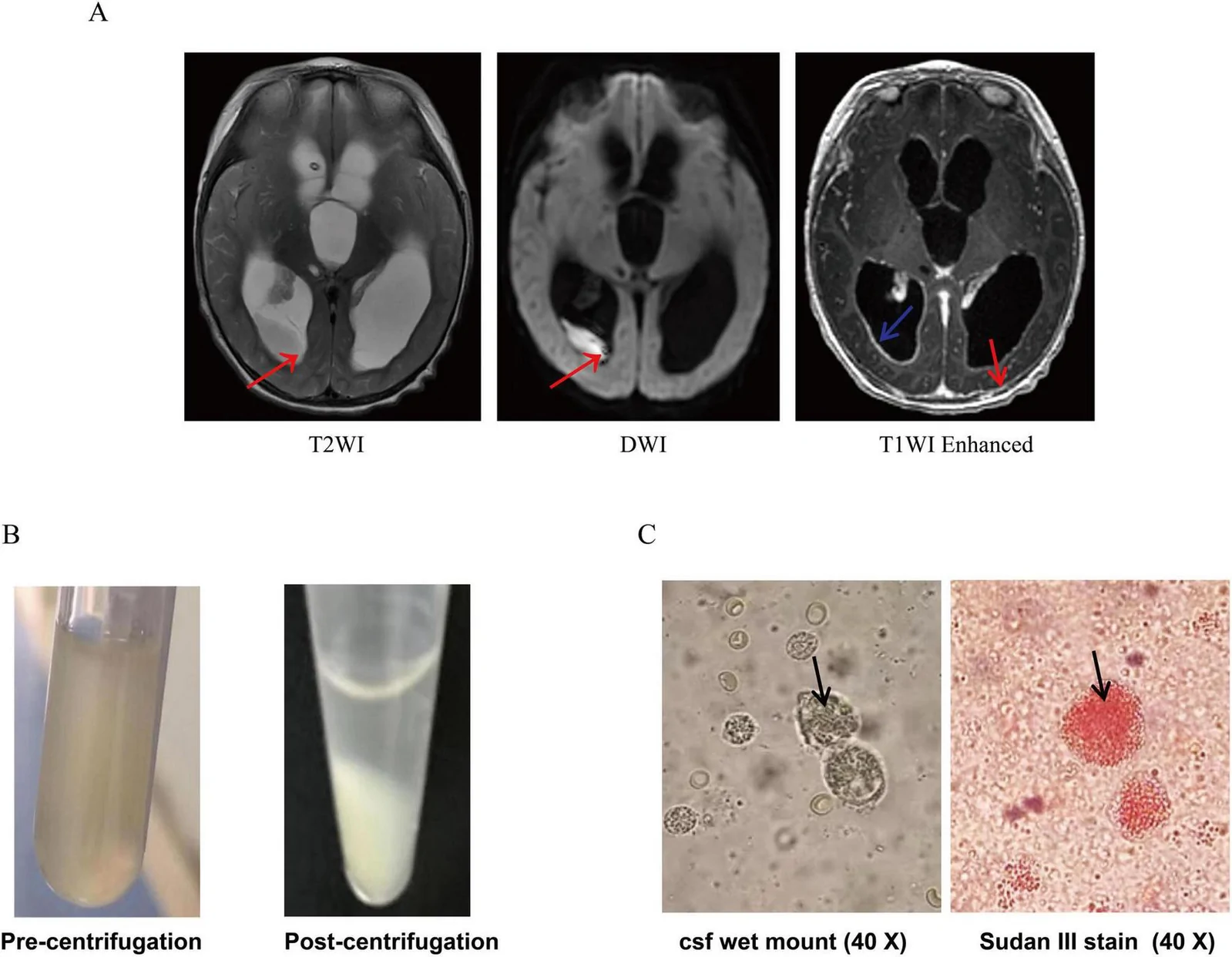
Index case of pediatric purulent meningitis with lipid-containing cerebrospinal fluid. **(A)** Brain MRI performed on March 1st. Representative axial T2-weighted (T2WI), diffusion-weighted (DWI), and post-contrast T1-weighted (T1WI Enhanced) images show abnormal intracranial lesions; red arrows indicate representative lesion sites. **(B)** Macroscopic appearance of the index CSF sample. Left: freshly collected CSF showing a strawberry-milk-like yellow and turbid appearance. Right: the same CSF sample after centrifugation at 12,000 rpm for 30 min using a refrigerated high-speed centrifuge, showing separation into a colorless, slightly turbid supernatant and a yellow-tinged sediment. **(C)** Microscopic examination of the CSF sample under a 40× objective. Left: CSF wet-mount preparation. Right: Sudan III-stained CSF smear showing lipid droplets (black arrows).

On March 3rd, the patient developed a sudden relapse of high fever (peak 39 °C), and CSF cultures grew *Pseudomonas aeruginosa*. Meropenem and amikacin were administered based on antimicrobial susceptibility testing and proved effective. Another bilateral ventricular puncture and drainage were performed on March 11th to alleviate intracranial hypertension, with plans for an Ommaya reservoir or ventriculoperitoneal shunt to address hydrocephalus. To improve cerebral infection control, intraventricular administration of “6 mg amikacin in 3 mL saline” was conducted under strict aseptic conditions. Despite effective temperature control and a notable reduction in white blood cell counts with meropenem and amikacin, repeated CSF cultures between March 3rd and March 14th continued to identify *Pseudomonas aeruginosa*. Magnetic resonance imaging on March 1st showed bilateral enlargement of the lateral, third, and fourth ventricles, periventricular edema, and aberrant brainstem morphology; contrast-enhanced imaging revealed localized thickening and enhancement of the meninges with small periventricular ring-enhancements adjacent to the anterior part of the lateral ventricles (Figure 1A). CSF analysis showed a further increase in white blood cell count (>1000 × 10^6^/L), elevated protein, and significantly reduced glucose (Table 2).

**TABLE 2:** Laboratory findings and lipid measurements of the representative index case with lipid-containing CSF.

Sample (collection date)	WBC	RBC	NEU	LYM	MON	CL	GLU	TP	TC	TG	HDL	LDL
Blood (Mar 14th)	8860	4200	5060	2070	1040	/	/	/	3.70	0.80	1.19	1.87
CSF (Mar 14th)	8323	12	6742	8.32	1498	111.9	1.63	4.27	0.21	0.05	0.03	0.01
CSF (Mar 16th)	72	0.3	26	33	13	108.2	2.06	2.19	/	/	/	/

Units: RBC × 10^9^/L; WBC, NEU, LYM, and MON × 106/L; CL, GLU, TC, TG, HDL, and LDL in mmol/L; TP in g/L. Reference interval for CSF total protein in our laboratory is 0.05–0.45 g/L. This table summarizes laboratory findings from the representative lipid-containing index case only and does not define the full study group allocation. Patient grouping and sample-level lipidomic allocation are described in Table 1 and the Methods section.

Notably, the CSF drained on March 14th exhibited a strawberry-milk-like yellow appearance with turbidity (Figure 1B). Routine CSF testing of this index sample showed marked inflammatory abnormalities, including a total protein level of 4.27 g/L, substantially above the laboratory reference interval of 0.05–0.45 g/L. After centrifugation at 12,000 rpm for 30 min using a refrigerated high-speed centrifuge, the CSF supernatant appeared colorless with slight turbidity, while the sediment exhibited a subtle yellow tint (Figure 1B). The CSF supernatant contained measurable lipid components (TC 0.21 mmol/L, TG 0.05 mmol/L, HDL 0.03 mmol/L, LDL 0.01 mmol/L). In contrast, the same-day blood lipid profile was within the pediatric reference range in our laboratory (TC 3.70 mmol/L, TG 0.80 mmol/L, HDL 1.19 mmol/L, LDL 1.87 mmol/L) (Table 2). The contrast between detectable CSF lipids and a normal blood lipid profile suggests that the lipid-containing appearance of this CSF sample was unlikely to be explained solely by systemic hyperlipidemia. Sudan III staining of the CSF smears revealed distinct lipid droplets (Figure 1C). On March 16th, the CSF total protein level decreased to 2.19 g/L, although it remained above the reference interval (Table 2). Review of the available clinical records did not identify systemic hyperlipidemia or documented parenteral lipid emulsion administration around the time of this index CSF sampling.

### Overview of CSF lipidomic profiles across N, PM_A, PM_R, and VM_A groups

**FIGURE 2:**
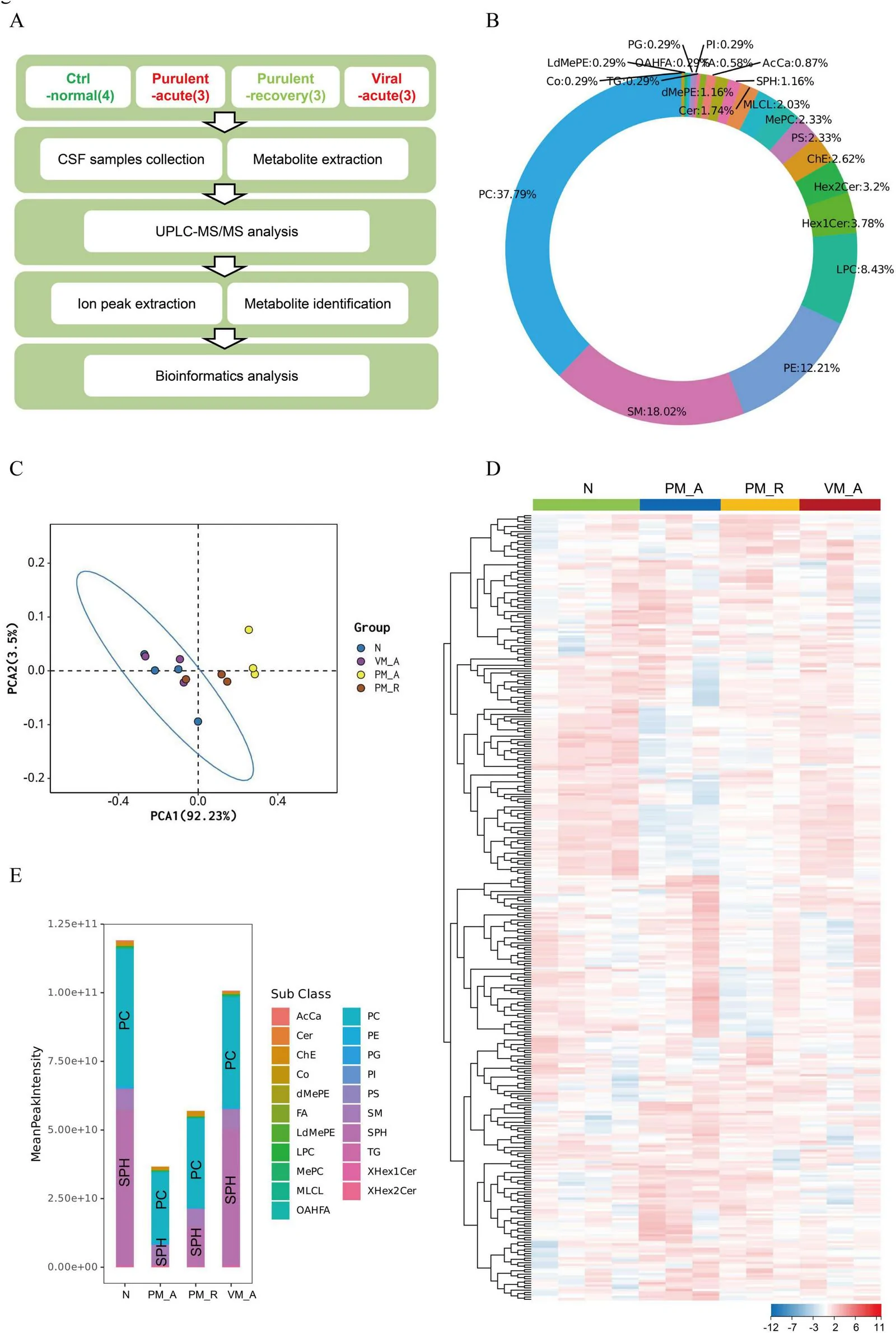
Lipidomic overview of CSF samples across N, PM_A, PM_R, and VM_A groups. **(A)** Workflow of comprehensive lipidomic analysis. **(B)** Composition of lipid sub-classes among the 344 identified lipid metabolites. **(C)** Principal component analysis (PCA) of the four analytical groups. **(D)** Heatmap of lipid metabolites across groups. **(E)** Stacked bar plot showing the mean peak intensity of lipid sub-classes. SPH and PC, the predominant sub-classes, are labeled within the bars for clarity; remaining sub-classes are shown in the legend.

To explore the relationship between CSF lipid composition and pediatric meningitis, 13 CSF samples from 10 pediatric patients (3 PM with paired acute- and recovery-phase CSF, 3 VM with acute-phase CSF only, and 4 N controls) were analyzed across four sample-level analytical groups: PM_A (*n* = 3), PM_R (*n* = 3), VM_A (*n* = 3), and N (*n* = 4). Paired acute- and recovery-phase samples were available only for PM patients, whereas VM patients contributed acute-phase samples only. Comprehensive lipidomic analysis using UPLC-MS/MS (Figure 2A) identified 344 lipid metabolites across 21 sub-classes and 6 lipid categories. These included 130 phosphatidylcholines (PC, 37.79%), 62 sphingomyelins (SM, 18.02%), 42 phosphatidylethanolamines (PE, 12.21%), and 29 lysophosphatidylcholines (LPC, 8.43%), among others (Figure 2B; lipid category and main-class composition across the four analytical groups is shown in Supplementary Figure 1B). Principal component analysis showed partial separation among groups, with PM_A samples tending to cluster separately from N, PM_R, and VM_A samples (Figure 2C). Heatmap analysis also suggested distinct lipidomic patterns across groups (Figure 2D). Because of the small sample size, these clustering patterns were interpreted as exploratory rather than confirmatory. Figure 2E provides a descriptive overview of lipid-class mean peak intensities across groups; the stacked bar plot was dominated by SPH- and PC-related signals, which are labeled within the bars for visual clarity, while lower-abundance subclasses are summarized numerically in Table 3, with representative high-intensity lipid classes per group and their exploratory biomarker relevance highlighted in Table 4. Fold changes and nominal exploratory *P*-values relative to the N group are provided in Supplementary Table 1. To avoid overinterpretation, lipid-class differences were interpreted descriptively only.

**TABLE 3:** Descriptive mean peak intensity of main lipid classes across analytical groups.

Lipid class	N	PM_A	PM_R	VM_A
AcCa	4.21 × 10^8^	2.12 × 10^7^	8.81 × 10^7^	3.40 × 10^8^
Cer	1.73 × 10^7^	1.25 × 10^7^	1.15 × 10^7^	1.42 × 10^7^
ChE	1.64 × 10^9^	1.25 × 10^9^	1.93 × 10^9^	8.15 × 10^8^
Co	1.84 × 10^7^	5.15 × 10^6^	8.43 × 10^6^	1.17 × 10^7^
FA	2.18 × 10^7^	1.02 × 10^8^	3.80 × 10^7^	6.17 × 10^7^
Hex1Cer	1.69 × 10^8^	1.36 × 10^8^	1.24 × 10^8^	1.51 × 10^8^
Hex2Cer	1.07 × 10^8^	4.83 × 10^8^	1.14 × 10^8^	1.33 × 10^8^
LPC	3.11 × 10^8^	3.37 × 10^8^	3.54 × 10^8^	3.18 × 10^8^
LdMePE	1.02 × 10^5^	3.83 × 10^5^	6.70 × 10^5^	1.06 × 10^5^
MLCL	1.08 × 10^7^	1.20 × 10^7^	1.96 × 10^7^	1.13 × 10^7^
MePC	6.28 × 10^8^	1.68 × 10^8^	1.93 × 10^8^	5.62 × 10^8^
OAHFA	9.62 × 10^4^	4.06 × 10^5^	8.02 × 10^4^	6.99 × 10^5^
PC	4.99 × 10^10^	2.54 × 10^10^	3.25 × 10^10^	4.02 × 10^10^
PE	1.01 × 10^9^	1.07 × 10^9^	4.86 × 10^8^	7.13 × 10^8^
PG	1.43 × 10^5^	4.33 × 10^5^	1.78 × 10^5^	3.06 × 10^4^
PI	2.27 × 10^6^	2.08 × 10^6^	2.49 × 10^6^	3.72 × 10^6^
PS	2.40 × 10^8^	9.18 × 10^7^	1.20 × 10^8^	1.45 × 10^8^
SM	7.56 × 10^9^	7.08 × 10^9^	6.59 × 10^9^	7.18 × 10^9^
SPH	5.64 × 10^10^	3.77 × 10^8^	1.42 × 10^10^	4.95 × 10^10^
TG	6.28 × 10^8^	6.59 × 10^7^	1.97 × 10^8^	5.48 × 10^8^
dMePE	3.16 × 10^7^	2.65 × 10^7^	1.48 × 10^7^	2.41 × 10^7^

Values are presented as mean peak intensity in scientific notation. Because each group contained only a small number of samples, this table is intended as a descriptive summary of lipid-class abundance rather than a confirmatory statistical comparison. Fold changes and nominal exploratory *P*-values for comparisons with the N group are provided in Supplementary Table 1 and should be interpreted as descriptive and hypothesis-generating only. N, non-meningitic control; PM_A, acute-phase purulent meningitis; PM_R, recovery-phase purulent meningitis; VM_A, acute-phase viral meningitis; AcCa, acylcarnitines; Cer, ceramide; ChE, cholesterol ester; Co, coenzyme; dMePE, dimethylphosphatidylethanolamine; FA, fatty acid; Hex1Cer, monohexosylceramide; Hex2Cer, dihexosylceramide; LdMePE, lysodimethylphosphatidylethanolamine; LPC, lysophosphatidylcholine; MePC, methylphosphocholine; MLCL, monolysocardiolipin; OAHFA, omega-acyl-hydroxy fatty acid; PC, phosphatidylcholine; PE, phosphatidylethanolamine; PG, phosphatidylglycerol; PI, phosphatidylinositol; PS, phosphatidylserine; SM, sphingomyelin; SPH, sphingolipid/sphingosine-related lipids; TG, triglyceride.

**TABLE 4:** Representative high-intensity lipid classes by group and exploratory biomarker relevance.

Group	Top 3 lipid classes by mean peak intensity	Potential biomarker implication
N	SPH (5.64 × 10^10^); PC (4.99 × 10^10^); SM (7.56 × 10^9^)	Reference baseline
PM_A	PC (2.54 × 10^10^); SM (7.08 × 10^9^); SPH (3.77 × 10^8^)	Hex2Cer/SPH/LPC axis prioritized for PM_A; LPC(20:3) and Hex2Cer(d42:3) candidate features
PM_R	PC (3.25 × 10^10^); SPH (1.42 × 10^10^); SM (6.59 × 10^9^)	Partial restoration toward N pattern
VM_A	SPH (4.95 × 10^10^); PC (4.02 × 10^10^); SM (7.18 × 10^9^)	SM/SPH/PS/MePC/LPC candidates remain exploratory; SM(d35:2) candidate feature

Lipid classes were ranked according to mean peak intensity within each analytical group based on Table 3. A separate VM_R group was not included because recovery-phase VM samples were not available. All biomarker implications are exploratory and require validation in larger pediatric cohorts.

### Exploratory lipid alterations in acute- and recovery-phase PM CSF

**FIGURE 3:**
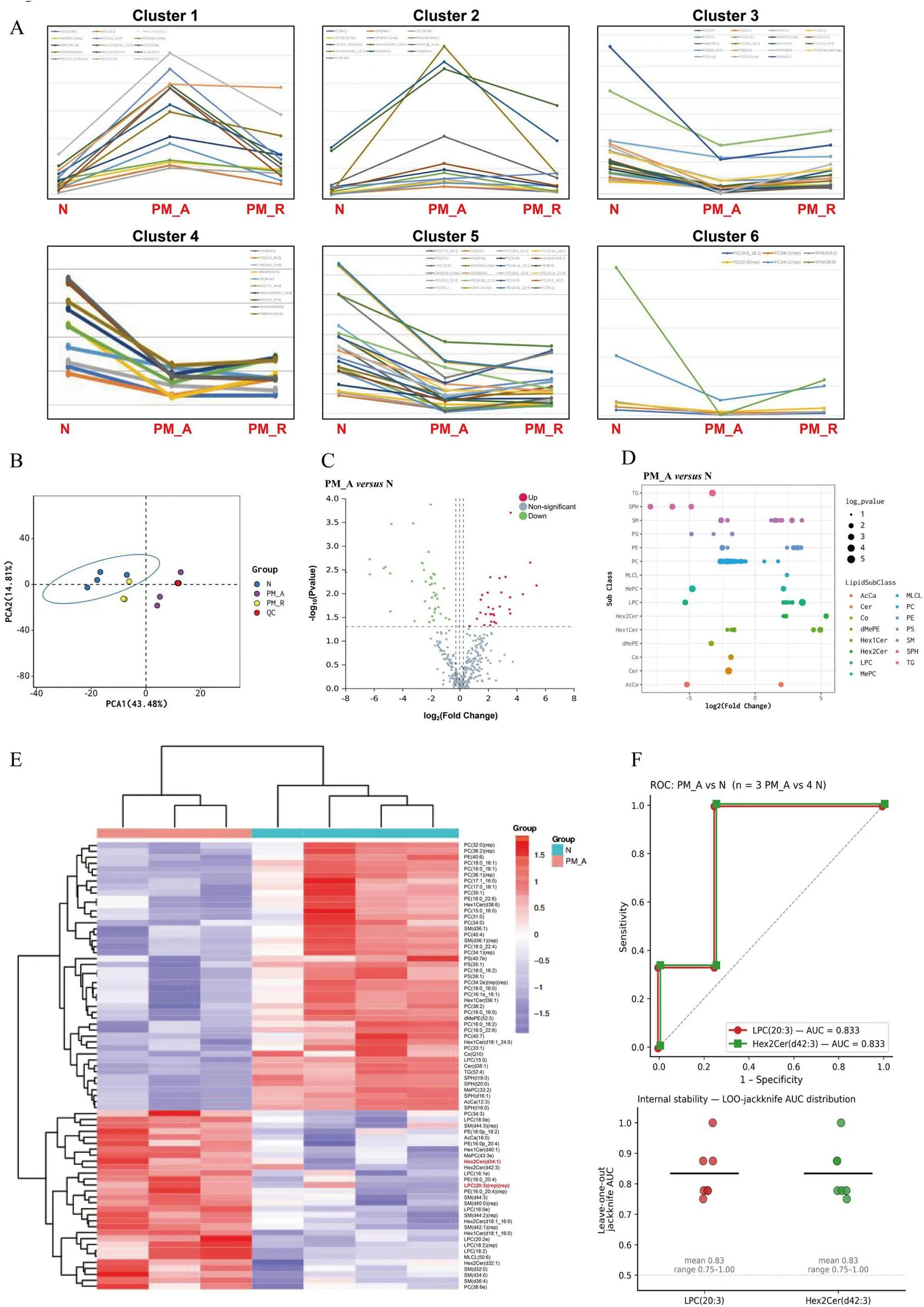
Exploratory lipid alterations in PM CSF. **(A)** Cluster categorization of differentially regulated lipid metabolites across the PM_A, PM_R, and N groups based on whether they returned to N levels at recovery, with mean peak intensity profiles. **(B)** PCA of the N, PM_A, and PM_R groups. The distribution of differentially regulated lipid metabolites between N and PM_A is shown by panel **(C)** volcano plot, **(D)** bubble plot, and **(E)** heatmap. **(F)** Receiver operating characteristic (ROC) analysis for the candidate PM_A vs. N markers. Top panel: ROC curves for LPC(20:3) (apparent AUC = 0.833) and Hex2Cer(d42:3) (apparent AUC = 0.833) distinguishing PM_A (*n* = 3) from N (*n* = 4) samples. Markers indicate the discrete points generated by the small sample size; the dashed diagonal indicates random performance. Bottom panel: leave-one-out (LOO) jackknife AUC distributions for each feature, with horizontal lines indicating mean AUC and labeled mean ± range. Single-feature jackknife AUCs ranged from 0.750 to 1.000 (mean 0.833) for both markers, indicating substantial sensitivity to single-sample removal and consistent with the limited statistical power inherent to a 3 vs. 4 comparison. Multi-feature composite ROC scores were intentionally not computed because, at *n* = 7, any data-driven combination would be highly susceptible to overfitting. All AUC values represent within-cohort performance only and require validation in independent pediatric cohorts. Hex2Cer(d42:3) was selected for ROC analysis based on the original LipidSearch/vendor differential analysis pipeline; other Hex2Cer species [e.g., Hex2Cer(d34:1), highlighted in panel **(E)**] showed similar between-group trends and are reported in the Supplementary Table 3.

Among comparisons involving the PM_A, PM_R, and N groups, 109 lipid metabolites met the exploratory screening criteria (FC ≥ 1.2 or ≤1/1.2 and nominal *P* < 0.05; Figure 3A, Supplementary Figure 2A). The numbers of differentially regulated lipid metabolites in the four pairwise comparisons (PM_A vs. N, PM_R vs. PM_A, VM_A vs. N, and VM_A vs. PM_A) are summarized in Supplementary Figure 1A. These metabolites were categorized according to their dynamic changes from the acute to the recovery phase. Compared with the N group, the PM_A group showed an exploratory lipid pattern characterized by markedly reduced SPH-related signals and increased Hex2Cer-related signals. For example, selected Hex2Cer species increased during the acute phase and tended to decrease toward the recovery phase, whereas several SPH-related species were reduced during the acute phase and showed only partial restoration during recovery (Figure 3A, Clusters 3–6). These findings suggest that altered sphingolipid-related metabolism may be associated with acute purulent meningitis, although this observation should be considered hypothesis-generating because of the limited sample size.

Principal component analysis of the N, PM_A, and PM_R groups showed partial separation, with PM_A samples tending to cluster distinctly from N and PM_R (Figure 3B). In the comparison of PM_A vs. N, 30 lipid metabolites were increased and 45 decreased according to the exploratory screening criteria, involving SPH, Hex2Cer, Hex1Cer, LPC, and AcCa subclasses (Figures 3C–E). Representative increased species included LPC(16:0e) (FC = 11.96), Hex1Cer(d18:1_16:0) (FC = 30.88), PE(16:0_20:4) (FC = 9.31), and several Hex2Cer species, while representative decreased species included Cer(d38:1) (FC = 0.25), TG(52:4) (FC = 0.11), MePC(33:2) (FC = 0.04), SPH(t18:0) (FC = 0.013), and SPH(t16:0) (FC = 0.004). To address multiplicity, BH-FDR *Q*-values were calculated and reported in the supplementary per-metabolite statistics (Supplementary Table 2); 4 metabolites achieved Q < 0.05 [Cer(d38:1), LPC(16:0e), TG(52:4), and MePC(33:2)] and a total of 29 metabolites achieved Q < 0.10 in the PM_A vs. N comparison (Supplementary Figure 3), indicating substantial statistical signal in this comparison despite the very small sample size. ROC analysis identified LPC(20:3) and Hex2Cer(d42:3) as exploratory candidate features for distinguishing PM_A from N, with an apparent AUC of 0.833 for each marker (Figure 3F). Internal stability was further explored using leave-one-out (LOO) jackknife: the LOO-jackknife AUCs ranged from 0.750 to 1.000 (mean 0.833) for each marker, indicating that a single sample swap could shift the AUC by up to one quartile in this small cohort. Multi-feature composite ROC scores (e.g., sum-of-z-score combinations) were not reported because, at *n* = 7 samples per comparison, any data-driven combination is highly susceptible to overfitting and would yield artificially perfect AUC values that do not reflect true generalizable performance. Because no independent validation cohort was available, these results were interpreted as exploratory within-cohort performance estimates only. In the PM_R vs. N comparison, 20 lipid metabolites were increased [e.g., several PC ether-linked species such as PC(38:6e), PC(40:7e), and SM(d42:1)] and 46 were decreased [e.g., PE(40:6), Cer(d38:1), LPC(20:2e); Supplementary Figure 2B and Supplementary Table 3]. For the recovery-versus-acute change, the figure (Supplementary Figures 2C,D) plots the PM_A vs. PM_R direction. Equivalently, in the PM_R vs. PM_A direction, 25 metabolites were increased in PM_R relative to PM_A [most prominently SPH(t16:0) and MePC(33:2), reflecting partial restoration of these species during recovery] and 17 were decreased in PM_R relative to PM_A [most prominently Hex2Cer(d32:1), Hex1Cer(d18:1_16:0), and LPC(20:2e)], suggesting partial remodeling of the CSF lipid profile during recovery. The recovery-phase pattern was characterized descriptively by partial restoration of SPH-related signals and reduction of selected LPC- and Hex2Cer-related alterations compared with the acute phase. These findings support the hypothesis that CSF lipid profiles may change dynamically during PM progression and recovery, but larger longitudinal cohorts are required for validation.

### Exploratory lipid features in acute-phase VM CSF

To describe lipidomic features associated with acute-phase viral meningitis, the VM_A group was analyzed separately, with the comparator clearly defined for each analysis. Compared with the N group, VM_A showed only 4 increased and 6 decreased metabolites meeting the exploratory screening criteria (Supplementary Figures 2E,F), suggesting that the overall lipidomic deviation of VM_A from N was less pronounced than that observed in PM_A. In the VM_A vs. PM_A comparison, 32 metabolites were increased and 15 decreased relative to PM_A (Supplementary Figures 2G,H show the equivalent PM_A vs. VM_A direction), involving SPH, PS, MePC, LPC, and AcCa sub-classes. Among the most prominent increased species (relative to PM_A) were MePC(33:2) (FC = 27.41), TG(52:4) (FC = 8.31), SPH(t16:0) (FC = 216.45), PC(18:0_18:1) (FC = 3.58), and SM(d36:1) (FC = 4.78). Among the most prominent decreased species (relative to PM_A) were PC(36:3) (FC = 0.24), LPC(18:0e) (FC = 0.08), MePC(43:3e) (FC = 0.14), SM(d36:4) (FC = 0.41), and PC(38:6e) (FC = 0.15). The directions of change in this comparison should not be interpreted as VM_A changes relative to N; rather, these patterns reflect how VM_A differs from PM_A and largely mirror the inverse of the PM_A-versus-N alterations (e.g., several lipid species reduced in PM_A relative to N appear increased in VM_A relative to PM_A). For VM_A vs. N specifically, only 4 metabolites were increased [FA(22:5), OAHFA(36:3), SM(d33:1), PC(30:0e)] and 6 were decreased [PC(38:2), SM(d35:2), PS(39:1), PC(18:0_18:2), PS(40:7e), and one additional PE species], under the exploratory screening criteria. ROC analysis identified SM(d35:2) as an exploratory candidate feature distinguishing VM_A from both N and PM_A, with apparent AUC = 1.000 and LOO-jackknife AUC = 1.000 across all folds in both comparisons; this candidate requires validation in larger cohorts. Because recovery-phase VM samples were not available, the VM-related findings in this study are limited to the acute phase.

## Discussion

Lipid abnormalities are increasingly recognized as part of the host metabolic response to central nervous system (CNS) infection and inflammation. In the present proof-of-concept case series, we observed a lipid-containing CSF appearance in a pediatric patient with severe purulent meningitis and identified distinct CSF lipidomic patterns among non-meningitic controls, acute purulent meningitis, recovery-phase purulent meningitis, and acute viral meningitis. These findings suggest that CSF lipid profiling may provide complementary information on infection-associated neuroinflammatory states, although the small sample size precludes definitive biomarker validation.

The grossly lipid-containing CSF observed in the index case should be interpreted in the context of both historical reports and recent CSF lipidomics studies. Earlier case reports have described lipid-rich CSF in association with cerebral hemorrhage (Younes et al., 1974), systemic hyperlipidemia (Burke et al., 1981), or strawberry-milk-like CSF appearance (Su et al., 2020), indicating that visible lipid accumulation in CSF can occur under selected pathological conditions. A distinct CSF lipid signature has previously been reported in subarachnoid hemorrhage–induced posthemorrhagic hydrocephalus (Toft-Bertelsen et al., 2023); our index case, which presented with concomitant subarachnoid hemorrhage and hydrocephalus, may share elements of that compartment-specific signature in addition to the infection-driven lipid changes characterized in this cohort. However, recent metabolomics and lipidomics studies suggest that CSF lipid alterations in meningitis may represent more than passive entry of circulating lipids into the CSF compartment. de Araujo et al. (2020) reported that the phosphatidylcholine species PC ae C44:6 was significantly elevated in CSF from patients with bacterial meningitis and showed discriminatory value compared with other neurological diagnoses, and proposed that elevated CSF PC ae C44:6 may reflect ongoing CNS cell membrane stress or damage rather than inflammation alone. Al-Mekhlafi et al. (2021) further demonstrated that increased free CSF phosphatidylcholines can distinguish bacterial meningitis from viral CNS infections and controls, supporting the concept that bacterial meningitis is associated with a disease-specific shift in CSF lipid metabolism.

Viral CNS infections may also display distinct CSF metabolic and lipidomic signatures. Ratuszny et al. (2019) identified CSF metabolites associated with enteroviral meningitis, supporting the value of CSF metabolomics in viral meningitis characterization. In a targeted metabolomics study, Al-Mekhlafi et al. (2023) reported elevated CSF phospholipids and short-chain acylcarnitines (C4 and C5) in viral CNS infections compared with autoimmune neuroinflammation, indicating that viral infection is also accompanied by compartment-specific lipid and energy-metabolism alterations. More recently, Neu et al. (2024) described CSF metabolite signatures differentiating viral encephalitis/meningitis from COVID-19-associated neurological involvement and aseptic neuroinflammation; notably, the triglyceride species TG(20:1_32:3) was higher in viral CNS infection than in neuro-COVID groups. Collectively, these studies demonstrate that CSF lipid and metabolite alterations differ across bacterial meningitis, viral CNS infection, and non-inflammatory or non-meningitic controls.

Our findings are broadly consistent with this emerging literature but also highlight several pediatric and disease-stage-specific observations. In the acute purulent meningitis group, we observed a marked reduction in SPH-related signals together with increased Hex2Cer-related signals and changes in LPC and acylcarnitine species. Although our cohort is too small for definitive diagnostic claims, the Hex2Cer/SPH/LPC axis may represent an exploratory lipid pattern associated with acute purulent meningitis. The reduction in SPH and elevation of Hex2Cer may reflect altered sphingolipid metabolism, membrane remodeling, inflammatory cell turnover, or tissue injury within the CNS compartment. In contrast, the acute viral meningitis group showed a different pattern involving SPH, PS, MePC, LPC, AcCa, and SM-related changes, which is compatible with prior reports that viral CNS infections exhibit lipid and energy-metabolism signatures distinct from bacterial meningitis (Ratuszny et al., 2019; Al-Mekhlafi et al., 2023; Neu et al., 2024). Consistent with these observations, Czepiel et al. (2019) reported that CSF n-3 polyunsaturated fatty acids are reduced and selected long-chain monounsaturated fatty acids are increased in both bacterial and viral meningitis relative to controls, suggesting compartmentalized fatty-acid remodeling that mirrors the FA-class trends seen in the present cohort.

Mechanistically, elevated or redistributed CSF lipid species in meningitis should not be interpreted simply as blood lipid infiltration into CSF. Several mechanisms may contribute, including perturbation of neuronal, glial, inflammatory-cell, endothelial, or microbial membranes; cytopathic effects caused by infection; inflammatory cell recruitment and turnover; local CNS metabolic remodeling; impaired CSF clearance; and blood–brain or blood–CSF barrier dysfunction. This interpretation is particularly relevant to our index case, in which the blood lipid profile was within the pediatric reference range while the CSF sample showed measurable lipid components and Sudan III–positive lipid droplets. Moreover, plasma lipid alterations may not mirror CSF lipid changes; previous work suggests that plasma phospholipids may decrease in bacterial meningitis owing to systemic inflammation, whereas selected CSF phospholipid species may increase (de Araujo et al., 2020; Al-Mekhlafi et al., 2021). CSF lipid abnormalities may therefore reflect a compartmentalized CNS response rather than a direct reflection of circulating lipid levels. Compartment-specific CSF lipid changes that do not mirror the plasma lipidome have also been observed in non-infectious pediatric neurological disease (Zandl-Lang et al., 2022).

Several specific molecular mechanisms may help account for the lipid pattern observed in the acute purulent meningitis (PM_A) group, and we propose them here as candidate hypotheses for future testing rather than as established conclusions. First, the marked elevation of selected ether-linked and polyunsaturated lysophosphatidylcholine (LPC) species – most prominently LPC(16:0e) (FC ≈ 12, BH-FDR Q < 0.05) and LPC(20:3), the latter identified as an exploratory ROC candidate (apparent AUC = 0.833) – is biochemically consistent with phospholipase A2 (PLA2)–mediated hydrolysis of membrane phosphatidylcholine, which generates LPC and free fatty acid; PLA2 isoforms have been broadly implicated in CNS inflammation. The observation that aggregate LPC subclass abundance is essentially unchanged in PM_A (FC ≈ 1.08) while individual LPC species are markedly altered is consistent with selective release of particular acyl chains rather than generalized phospholipid lipolysis. Second, the simultaneous depletion of sphingoid base (SPH) signal (subclass FC ≈ 0.007 vs. N) and accumulation of dihexosylceramide (Hex2Cer) species (subclass FC ≈ 4.5; the species Hex2Cer(d18:1_16:0) is increased ≈ 42-fold in PM_A vs. N) is not parsimoniously explained by direct sphingomyelinase-mediated SM hydrolysis, which would instead predict reduced sphingomyelin and elevated ceramide; in our cohort, SM (FC ≈ 0.94) and total Cer (FC ≈ 0.72) are essentially preserved at the subclass level. The observed SPH↓/Hex2Cer↑ pattern is more consistent with altered sphingoid-base flux into the glycosphingolipid biosynthetic pathway (UDP-sugar–dependent stepwise glucosyl/galactosyl transfer onto a ceramide backbone), or alternatively with selective pathogen-driven scavenging or remodeling of host sphingolipid pools. Third, both *Streptococcus pneumoniae* and *Pseudomonas aeruginosa* – the two pathogens identified in the index case – possess documented host-lipid–remodeling capacities, including pneumococcal phosphorylcholine surface decoration (Maestro and Sanz, 2016) and pseudomonal phospholipase/lipase virulence factors (Wolfmeier et al., 2022). The PM_A CSF lipid signature observed here may therefore reflect the convergent product of host inflammatory lipase activation and direct pathogen-driven lipid remodeling, rather than a single dominant mechanism. Direct testing of these hypotheses will require paired CSF/serum lipidomics with Q-albumin measurement, CSF sPLA2 and sphingomyelinase activity assays, and pathogen-specific *in vitro* lipid-remodeling experiments – directions we identify as priorities for future work.

These observations should nevertheless be interpreted cautiously. The present study was exploratory, with a limited number of pediatric CSF samples, and no independent validation cohort was available. To partially address this, we performed an internal LOO-jackknife procedure for the candidate biomarker features. Although LOO-jackknife AUCs are reported as a measure of internal stability, this procedure cannot substitute for external validation. Particular caution is warranted in interpreting markers with apparent within-cohort AUC = 1.000, such as SM(d35:2) for VM_A discrimination: in a comparison of 3 vs. 3 or 3 vs. 4 samples, simple combinatorial considerations show that a fraction of the 344 screened metabolites will show perfect separation by chance alone, irrespective of underlying biology. We therefore deliberately did not report multi-feature composite ROC scores (which would frequently yield artificially perfect AUC = 1.000 in this sample-size regime) and we frame all candidate markers, including SM(d35:2), as hypothesis-generating only. Paired acute- and recovery-phase CSF samples were available only for PM patients, whereas VM patients contributed only acute-phase CSF samples; therefore, recovery-phase VM lipid changes could not be assessed. The cohort did not include patients with tuberculous meningitis, and lipid features specific to pediatric tuberculous meningitis remain to be addressed in future studies; for context, prior CSF amino-acid metabolomics in pediatric TBM has highlighted distinct host-derived metabolic perturbations (Mason et al., 2017). In addition, routine lipid measurements were available for the visibly lipid-containing index CSF sample but not for all non–lipid-appearing follow-up samples, preventing direct paired comparison of routine CSF lipid concentrations. The study also did not include paired serum–CSF lipidomics, paired CSF/serum albumin measurements, or Q-albumin analysis [Q-albumin, the CSF/serum albumin ratio, is the standard marker of blood–CSF barrier permeability (Reiber and Peter, 2001)]. Therefore, the relative contributions of membrane injury, cytopathic effects, barrier dysfunction, impaired CSF clearance, and systemic lipid metabolism could not be disentangled. Markedly elevated CSF total protein (4.27 g/L) in the index case suggests substantial blood–CSF barrier disruption and/or intrathecal inflammatory exudation, but CSF total protein is non-specific and cannot replace Q-albumin. Future prospective studies with larger pediatric cohorts, paired serum and CSF samples, CSF albumin measurements, and longitudinal CSF lipid profiling are needed to validate these candidate lipid patterns and clarify their biological origin.

Overall, our study supports the potential of CSF lipidomics as a complementary tool for characterizing the pathophysiology of pediatric meningitis and for generating candidate diagnostic and prognostic markers, while emphasizing that all candidate features identified here are exploratory and require external validation.

## Data Availability

The raw data supporting the conclusions of this article will be made available by the authors, without undue reservation.

## Supplementary material

Supplementary Table 1 Lipid sub-class fold change and nominal exploratory *P*-value relative to the non-meningitic (N) group.

Supplementary Table 2 Top 30 lipid metabolites in the PM_A vs N comparison ranked by ascending nominal *P*-value (Welch’s *t*-test on log10-transformed peak intensity, with Benjamini-Hochberg FDR Q-value).

Supplementary Table 3 Per-metabolite Welch’s *t*-test results, fold change, and Benjamini-Hochberg false discovery rate (BH-FDR) Q-values for all 344 identified lipid metabolites across the five pairwise comparisons. This supplementary spreadsheet (Per_metabolite_stats.xlsx) contains six worksheets: five comparison-specific worksheets (PM_A_vs_N, PM_R_vs_N, VM_A_vs_N, PM_R_vs_PM_A, and VM_A_vs_PM_A), each reporting all 344 metabolites, and a Summary worksheet tabulating the number of differentially regulated metabolites per comparison under the exploratory screening criteria. For each metabolite, the comparison worksheets report: Metabolite name, Adduct, Sub Class, Lipid Category, group-level mean peak intensities, fold change (FC, ratio of the first group mean to the second group mean), log_2_FC, nominal Welch’s *t*-test *P*-value computed on log10-transformed sample-level peak intensities (P_Welch_log10), and Benjamini-Hochberg false discovery rate Q-value (FDR_BH) computed within each comparison across all 344 metabolites. All per-metabolite statistics are exploratory and hypothesis-generating given the small sample size (PM_A *n* = 3, PM_R *n* = 3, VM_A *n* = 3, N *n* = 4). BH-FDR, Benjamini Hochberg false discovery rate; FC, fold change; N, non-meningitic control; PM_A, acute-phase purulent meningitis; PM_R, recovery-phase purulent meningitis; VM_A, acute-phase viral meningitis.
