# Correction: Cerebrospinal fluid lipid profiles as exploratory biomarkers for pediatric meningitis: a proof-of-concept case series

**DOI:** 10.3389/fncel.2026.2012403

**Published:** 2026-10-09

**Authors:** Feng Tang, Changzhen Li, Ye Zeng, Wanjun Luo, Lei Xi, Xiaomei Wang

**Affiliations:** 1 Department of Laboratory Medicine, Wuhan Children's Hospital (Wuhan Maternal and Child Healthcare Hospital), Tongji Medical College, Huazhong University of Science & Technology, Wuhan, China; 2 Hospital-Acquired Infection Control Department, Wuhan Children's Hospital (Wuhan Maternal and Child Healthcare Hospital), Tongji Medical College, Huazhong University of Science & Technology, Wuhan, China

**Keywords:** cerebrospinal fluid, differential diagnosis, lipid metabolomics, mass spectrometry, pediatric meningitis

The **Ethics statement** was erroneously given as [2021R064, 2024R05-E2]. The correct **Ethics statement** is [2024R053-E01, 2024R050-E01].

The original version of this article has been updated.

