# Supplementary figures and images for: Cerebrospinal fluid lipid profiles as exploratory biomarkers for pediatric meningitis: a proof-of-concept case series
Source: Front Cell Neurosci. 2026 May 29;20:1816621. doi: 10.3389/fncel.2026.1816621 (PMC13259668; doi:10.3389/fncel.2026.1816621)

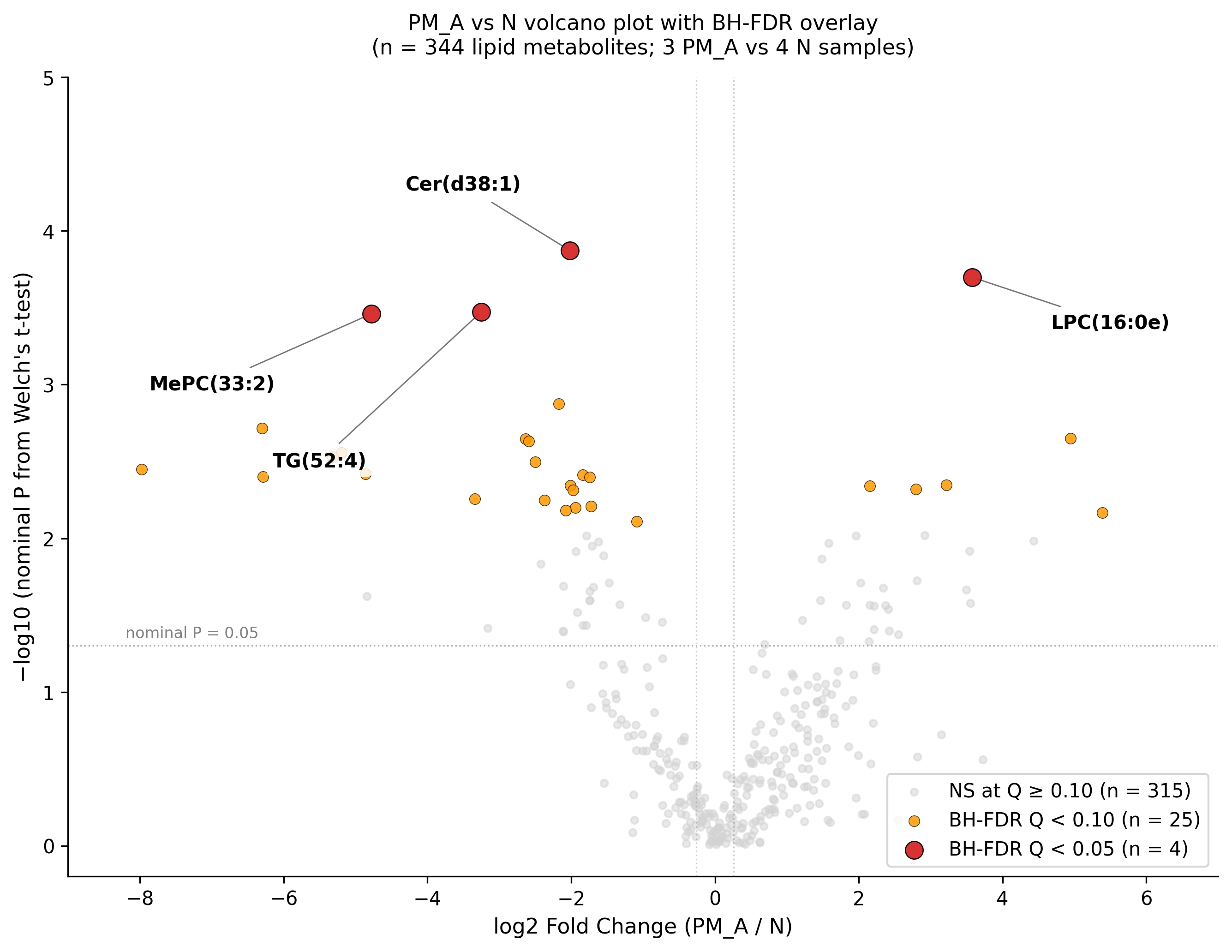

Supplement: Supplementary file 3 [file Image_3.jpeg]
