# Supplementary material for: Cerebrospinal fluid lipid profiles as exploratory biomarkers for pediatric meningitis: a proof-of-concept case series
Source: Front Cell Neurosci. 2026 May 29;20:1816621. doi: 10.3389/fncel.2026.1816621 (PMC13259668; doi:10.3389/fncel.2026.1816621)
Supplement: Supplementary Table 1 — Lipid sub-class fold change and nominal exploratory P-value relative to the non-meningitic (N) group. [file Table_1.DOCX]

**Supplementary Figure Legends**

**Supplementary Figure S1.** Differentially regulated lipid metabolites between groups and lipid composition overview. **(A)** Numbers of differentially regulated lipid metabolites in four pairwise comparisons (PM_A vs N: 30 increased / 45 decreased; PM_R vs PM_A: 25 increased / 17 decreased; VM_A vs N: 4 increased / 6 decreased; VM_A vs PM_A: 32 increased / 15 decreased), under the exploratory screening criteria of fold change ≥ 1.2 (or ≤ 1/1.2) and nominal P < 0.05. The PM_R vs N comparison (20 increased / 46 decreased) is reported in the main Results text and in the Per_metabolite_stats supplementary file but is not separately plotted here. **(B)** Composition of lipid categories and main classes among the 344 identified lipid metabolites across the four analytical groups.

**Supplementary Figure S2.** Detailed lipidomic comparisons between groups. **(A)** Additional cluster categorization (Clusters 7–10) of differentially regulated lipid metabolites across N, PM_A, and PM_R based on whether they returned toward N levels during recovery, with mean peak intensity profiles. **(B)** Volcano plot of PM_R vs N. **(C)** Volcano plot of PM_A vs PM_R. **(D)** Bubble plot of differentially regulated lipid sub-classes for PM_A vs PM_R. **(E)** Volcano plot of VM_A vs N. **(F)** Bubble plot of differentially regulated lipid sub-classes for VM_A vs N. **(G)** Volcano plot of PM_A vs VM_A. **(H)** Bubble plot of differentially regulated lipid sub-classes for PM_A vs VM_A. Throughout this figure, points or markers above the dashed line indicate exploratory screening criteria of FC ≥ 1.2 (or ≤ 1/1.2) and nominal P < 0.05. The directions of comparisons follow the original raw data analysis pipeline; in the main Results text, the equivalent reverse comparisons (e.g., PM_R vs PM_A and VM_A vs PM_A) are also referenced and the up- and down-regulated metabolite counts are reported with explicit comparator orientation.

**Supplementary Figure S3.** Volcano plot of the PM_A vs N comparison with BH-FDR overlay. Each point represents one of the 344 lipid metabolites identified in the lipidomic analysis. The x-axis is log2 fold change (PM_A divided by N), and the y-axis is −log10 of the nominal P value from Welch’s t-test on log10-transformed sample-level peak intensities. Points are colored by Benjamini–Hochberg false discovery rate (BH-FDR) Q value: light gray, Q ≥ 0.10 (n = 315); orange, 0.05 ≤ Q < 0.10 (n = 25); red, Q < 0.05 (n = 4). The four metabolites achieving Q < 0.05 are labelled (Cer(d38:1), MePC(33:2), TG(52:4), LPC(16:0e)). Dashed gray lines indicate nominal P = 0.05 and the FC ≥ 1.2 (or ≤ 1/1.2) screening criterion, both of which are exploratory and not confirmatory. The plot demonstrates that despite the very small sample size (3 PM_A vs 4 N), the PM_A vs N comparison yields a substantial number of metabolites with Q < 0.10 and four with Q < 0.05, consistent with a true acute-phase pediatric purulent meningitis lipid signature.
