# Supplementary material for: Cerebrospinal fluid lipid profiles as exploratory biomarkers for pediatric meningitis: a proof-of-concept case series
Source: Front Cell Neurosci. 2026 May 29;20:1816621. doi: 10.3389/fncel.2026.1816621 (PMC13259668; doi:10.3389/fncel.2026.1816621)
Supplement: Supplementary Table 2 — Top 30 lipid metabolites in the PM_A vs N comparison ranked by ascending nominal P-value (Welch’s t-test on log10-transformed peak intensity, with Benjamini-Hochberg FDR Q-value). [file Table_2.docx]

**Supplementary Table S1. Lipid sub-class fold change and nominal exploratory P value relative to the non-meningitic (N) group.**

| **Lipid sub-class** | **PM_A vs N** | | **PM_R vs N** | | **VM_A vs N** | |
| --- | --- | --- | --- | --- | --- | --- |
|  | **FC** | **Nominal P** | **FC** | **Nominal P** | **FC** | **Nominal P** |
| **AcCa** | 0.050 | **0.001** | 0.209 | **0.018** | 0.807 | 0.644 |
| **Cer** | 0.722 | 0.360 | 0.665 | 0.269 | 0.822 | 0.578 |
| **ChE** | 0.760 | 0.925 | 1.174 | 0.479 | 0.496 | 0.475 |
| **Co** | 0.279 | **0.004** | 0.457 | **0.036** | 0.634 | 0.211 |
| **dMePE** | 0.839 | 0.486 | 0.468 | 0.133 | 0.765 | 0.322 |
| **FA** | 4.653 | 0.293 | 1.742 | 0.439 | 2.827 | **0.049** |
| **Hex1Cer** | 0.803 | 0.467 | 0.732 | 0.227 | 0.892 | 0.547 |
| **Hex2Cer** | 4.502 | **0.017** | 1.059 | 0.617 | 1.239 | 0.783 |
| **LdMePE** | 3.770 | 0.113 | 6.603 | 0.147 | 1.044 | 0.855 |
| **LPC** | 1.083 | 0.973 | 1.136 | 0.849 | 1.020 | 0.795 |
| **MePC** | 0.267 | **0.016** | 0.307 | **0.016** | 0.895 | 0.731 |
| **MLCL** | 1.111 | 0.951 | 1.811 | 0.334 | 1.049 | 0.918 |
| **OAHFA** | 4.225 | 0.624 | 0.833 | 0.742 | 7.270 | **0.011** |
| **PC** | 0.510 | 0.057 | 0.651 | 0.097 | 0.805 | 0.432 |
| **PE** | 1.062 | 0.831 | 0.482 | 0.131 | 0.708 | **0.021** |
| **PG** | 3.034 | 0.706 | 1.246 | 0.846 | 0.215 | 0.324 |
| **PI** | 0.919 | 0.723 | 1.100 | 0.682 | 1.644 | 0.142 |
| **PS** | 0.383 | 0.053 | 0.500 | **0.024** | 0.604 | **0.042** |
| **SM** | 0.937 | 0.810 | 0.872 | 0.510 | 0.949 | 0.859 |
| **SPH** | 0.0067 | **0.002** | 0.252 | **0.042** | 0.878 | 0.739 |
| **TG** | 0.105 | **3.36×10⁻⁴** | 0.314 | **0.008** | 0.872 | 0.693 |

Lipid sub-class mean peak intensities were computed by summing all metabolite peak intensities within each sub-class for each sample, then averaging across samples within each analytical group (N = 4, PM_A = 3, PM_R = 3, VM_A = 3). Fold change (FC) is the ratio of the group mean (PM_A, PM_R, or VM_A) to the N mean. Nominal exploratory P values were computed by Welch's t-test on log10-transformed sample-level peak intensity sums; they are reported as descriptive and hypothesis-generating only and are NOT corrected for multiple testing across lipid sub-classes. Highlighted cells indicate nominal P < 0.05 (yellow). For Benjamini–Hochberg false discovery rate (BH-FDR) Q values across the 344 individual lipid metabolites, see Supplementary Table S2 (top 30 metabolites for the PM_A vs N comparison) and the Per_metabolite_stats supplementary spreadsheet (full results for all 5 pairwise comparisons). **Abbreviations.** AcCa, acylcarnitines; Cer, ceramide; ChE, cholesterol ester; Co, coenzyme; dMePE, dimethylphosphatidylethanolamine; FA, fatty acid; Hex1Cer, monohexosylceramide; Hex2Cer, dihexosylceramide; LdMePE, lysodimethylphosphatidylethanolamine; LPC, lysophosphatidylcholine; MePC, methylphosphocholine; MLCL, monolysocardiolipin; N, non-meningitic control; OAHFA, omega-acyl-hydroxy fatty acid; PC, phosphatidylcholine; PE, phosphatidylethanolamine; PG, phosphatidylglycerol; PI, phosphatidylinositol; PM_A, acute-phase purulent meningitis; PM_R, recovery-phase purulent meningitis; PS, phosphatidylserine; SM, sphingomyelin; SPH, sphingolipid/sphingosine-related lipids; TG, triglyceride; VM_A, acute-phase viral meningitis.

**Supplementary Table S2. Top 30 lipid metabolites in the PM_A vs N comparison ranked by ascending nominal P value (Welch's t-test on log10-transformed peak intensity, with Benjamini–Hochberg FDR Q value).**

| **Metabolite** | **Sub Class** | **Fold Change** | **log₂FC** | **Nominal P** | **BH-FDR Q** |
| --- | --- | --- | --- | --- | --- |
| Cer(d38:1) | Cer | 0.246 | -2.021 | 1.34×10⁻⁴ | **0.0298** |
| LPC(16:0e) | LPC | 11.962 | 3.580 | 2.00×10⁻⁴ | **0.0298** |
| TG(52:4) | TG | 0.105 | -3.252 | 3.36×10⁻⁴ | **0.0298** |
| MePC(33:2) | MePC | 0.0364 | -4.782 | 3.47×10⁻⁴ | **0.0298** |
| PC(18:0_18:1) | PC | 0.221 | -2.175 | 0.0013 | **0.0758** |
| SPH(t18:0) | SPH | 0.013 | -6.302 | 0.0019 | **0.0758** |
| Hex1Cer(d18:1_16:0) | Hex1Cer | 30.880 | 4.949 | 0.0022 | **0.0758** |
| PC(16:0_18:1) | PC | 0.161 | -2.635 | 0.0023 | **0.0758** |
| SM(d36:1)(rep) | SM | 0.166 | -2.591 | 0.0023 | **0.0758** |
| AcCa(12:3) | AcCa | 0.027 | -5.194 | 0.0028 | **0.0758** |
| LPC(15:0) | LPC | 0.025 | -5.319 | 0.0030 | **0.0758** |
| PE(40:6) | PE | 0.177 | -2.499 | 0.0032 | **0.0758** |
| SPH(t16:0) | SPH | 0.004 | -7.979 | 0.0036 | **0.0758** |
| SPH(d16:1) | SPH | 0.034 | -4.865 | 0.0038 | **0.0758** |
| Co(Q10) | Co | 0.279 | -1.842 | 0.0039 | **0.0758** |
| SPH(t20:0) | SPH | 0.013 | -6.289 | 0.0040 | **0.0758** |
| PC(36:1)(rep) | PC | 0.299 | -1.743 | 0.0040 | **0.0758** |
| PE(16:0_20:4) | PE | 9.312 | 3.219 | 0.0045 | **0.0758** |
| PC(16:0_16:0) | PC | 0.247 | -2.015 | 0.0045 | **0.0758** |
| MePC(43:3e) | MePC | 4.441 | 2.151 | 0.0046 | **0.0758** |
| SM(d42:1)(rep) | SM | 6.951 | 2.797 | 0.0048 | **0.0758** |
| PC(34:1)(rep) | PC | 0.254 | -1.976 | 0.0048 | **0.0758** |
| dMePE(52:5) | dMePE | 0.099 | -3.341 | 0.0055 | **0.0808** |
| PC(33:1) | PC | 0.193 | -2.371 | 0.0056 | **0.0808** |
| PC(32:0)(rep) | PC | 0.303 | -1.722 | 0.0062 | **0.0835** |
| PC(18:0_22:4) | PC | 0.260 | -1.945 | 0.0063 | **0.0835** |
| PC(17:0_18:1) | PC | 0.237 | -2.078 | 0.0066 | **0.0837** |
| Hex2Cer(d18:1_16:0) | Hex2Cer | 41.933 | 5.390 | 0.0068 | **0.0837** |
| PC(36:2)(rep) | PC | 0.470 | -1.091 | 0.0078 | **0.0923** |
| PE(16:0_20:4)(rep) | PE | 7.562 | 2.919 | 0.0095 | 0.1036 |

Per-metabolite Welch's t-test was performed on log10-transformed sample-level peak intensities (3 PM_A vs 4 N samples; handles unequal variances and is robust at small n). Fold change (FC) is the PM_A group mean divided by the N group mean. Benjamini–Hochberg (BH) false discovery rate (FDR) Q values were computed across all 344 metabolites in the PM_A vs N comparison. Cells highlighted in orange indicate Q < 0.05 (n = 4 metabolites: Cer(d38:1), LPC(16:0e), TG(52:4), MePC(33:2)); cells highlighted in yellow indicate 0.05 ≤ Q < 0.10 (n = 25 additional metabolites). The exploratory screening criterion of FC ≥ 1.2 (or ≤ 1/1.2) and nominal P < 0.05 used in the manuscript Results is descriptive and not a confirmatory significance threshold; BH-FDR Q values are reported here for transparency on multiple testing. Full per-metabolite results for all 5 pairwise comparisons (PM_A vs N, PM_R vs N, VM_A vs N, PM_R vs PM_A, VM_A vs PM_A) — covering all 344 metabolites — are provided in the accompanying Per_metabolite_stats spreadsheet.
